# Viruses of Plankton: On the Edge of the Viral Frontier

**DOI:** 10.3390/microorganisms12010031

**Published:** 2023-12-23

**Authors:** Kristina D. A. Mojica, Corina P. D. Brussaard

**Affiliations:** 1 Division of Marine Science, School of Ocean Science and Engineering, The University of Southern Mississippi, Stennis Space Center, Hancock County, MS 39529, USA; 2 Department of Marine Microbiology and Biogeochemistry, NIOZ—Royal Netherlands Institute for Sea Research, 1790 AB Den Burg, The Netherlands; corina.brussaard@nioz.nl; 3 Department of Freshwater and Marine Ecology, Institute for Biodiversity and Ecosystem Dynamics (IBED), University of Amsterdam, 1000 GG Amsterdam, The Netherlands

The field of aquatic viral ecology has continued to evolve rapidly over the last three decades. There are around 10^31^ virus particles on Earth [1,2], however, this remains a conservative estimate as small genome viruses are underrepresented in routine counting methods [3,4]. Viruses are a major source of mortality and disease in aquatic ecosystems, with an estimated 10^23^ viral infections occurring each second [5,6]. Most of these infections affect the numerically dominant plankton (i.e., prokaryotes, phytoplankton, and zooplankton) [5]. Lytic viral infection of aquatic microorganisms typically culminates with the lysis of the host to release viral progeny. Viral lysis liberates cellular material rich in organic matter and inorganic nutrients into the surrounding water, redirecting the flow of organic matter away from higher trophic levels towards microbial reprocessing, a process known as viral shunt (Figure 1; red arrows). It is estimated that virus-mediated mortality releases 10 billion tons of carbon per day [7,8], making viruses key players in global biogeochemical cycles. Still, ongoing discoveries highlight that many open questions remain regarding the complex and diverse roles that viruses play in planktonic food webs and how they are impacted by environmental factors. This Special Issue of *Microorganisms* provides key insights into our ever-evolving understanding of the ecology of planktonic viruses.

Viruses infect all living organisms, but in general, viruses infecting zooplankton are understudied [9]. This is primarily due to the scarcity of symptomatological studies of viral diseases in plankton and the absence of definitive links to host mortality in many marine species. The review in this Special Issue by Roberts & Suttle [9] provides an overview of the current knowledge of viral pathogens of crustacean zooplankton and their effects on marine food webs. The authors establish the plausibility for viral infection to be responsible for a significant portion of unexplained non-consumptive mortality of crustacean zooplankton and the potential for these hosts to serve as vectors of disease. Crustacean zooplankton and other carnivorous plankton can catalyze viral infection through their feeding activities. Small-sized zooplankton, such as small flagellates and ciliates, have also been reported to ingest (and digest) viruses [10,11,12] and therefore may act as a mechanism for viral removal and inactivation. However, from the viewpoint of viruses as prey, they have only been considered as a minor source of nutrition in the absence of preferred prey-bacteria. In this Issue, Al-Ameeli et al. [13] demonstrate the role that the ciliate *Bursaria truncatella* plays in exposing a chlorella-like green algae to viral infection as it feeds on its mutualistic endosymbiotic host *Paramecium bursaria*. Moreover, depending on the feeding mechanism and size of the predator, the efficiency with which the Chlorellavirus can replicate varies between 0.3% to 97%. Recent research confirms that ciliates possess ingestion rates sufficient to drive population growth [14] and enhance survival rates [15]. Significant prevalence of virovory in aquatic environments not only alters our view of carbon flow through marine food webs by introducing a new pathway for viruses to enter the food web (Figure 1; blue arrows; viral loop), but also modifies our conception of trophic interactions within food webs [16,17]. The articles by Roberts and Suttle [9] and Al-Ameeli et al. [13] expand upon our understanding of the myriad of ways in which viruses and zooplankton interact in aquatic environments (Figure 1; gray arrow and the viral loop).

Metagenomics has advanced our understanding of virus diversity in aquatic systems and illuminated the mechanisms through which viruses interact with their hosts [18]. One notable example is the ability of viruses to affect biogeochemical processes through the incorporation and expression of auxiliary metabolic genes (AMGs). Many virally encoded AMGs are involved in critical, rate-limiting steps of host metabolism and it is believed that they sustain or supplement host metabolism to maximize the production of new viral particles during infection [19,20]. The abundance of various AMGs within a community can reflect both niche-specific and temporal responses to environmental conditions and therefore provide information about the metabolic capacity of the microbial community and/or response to an environmental disturbance. In this Issue, Woods et al. [21] found that virally encoded metabolic genes associated with carbon, nitrogen, and sulfur metabolisms increased following Hurricane Harvey, suggesting that viruses may have aided in the recovery of the host community and thus play a vital role in ecosystem recovery after pulse disturbances. Potapov et al. [22] investigated viral metatranscriptomes and observed that, during the late spring season (July), viral AMGs for the metabolism of complex substrates, amino acids, cofactors, vitamins, and nucleotides were present in the oligotrophic Lake Baikal. This is contrast to the early spring (March–May) condition, when AMGs for carbohydrate metabolic pathways dominated concurrent with presumed high bacterial remineralization activity following the peak in phytoplankton production [23]. Potapov et al. also found that a number of viral transcripts encoded for ABC transporters, proteins that use ATP to transport substrates across membranes, which agrees with earlier findings suggesting membrane transport proteins may be common in virus genomes [24]. Most of the viral transport proteins that have been tested are functional and there is indirect evidence suggesting that they may be involved in various phases of the viral life cycle (i.e., viral penetration, replication, assembly, and release) [24]. Given the fact that most viral genes have not yet been characterized, studies like those by Potapov, Woods, and colleagues contribute to our understanding of the ways in which viruses manipulate host biology and physiology. 

Whilst our cognizance of planktonic viruses evolves, global climate change is affecting microbial host community composition, ecosystem functioning, and consequently virus–host interactions [25]. Climate change is increasing the intensity and frequency of climate-driven disturbances [16]. Physical disturbances play important roles in shaping biological landscapes [26,27], influencing ecosystem production [28], trophic interactions [29], and consequently carbon export [30,31] in the ocean. In this Issue, Woods et al. [21] reveals that salinity changes from heavy rainfall following the passage of Hurricane Harvey significantly affected the estuarine viral community located within Galveston Bay, decreasing evenness and richness. This was likely driven by a combination of the introduction of novel microbial hosts into the system and diluted viral concentration that ultimately reduced the frequency and success of virus–host contact rates. Additionally, the Southern Ocean and its regional seas are especially important to the global carbon cycle and are undergoing dramatic changes in response to warming conditions. It is thus imperative that more detailed studies on the loss rates of primary producers be conducted in this area, particularly with respect to viral infection rates, in order to fully understand the global consequences of these changes. In this Issue, Eich et al. [32] report that total phytoplankton loss rates were considerably higher inside compared to outside the warmer Amundsen Sea Polynya (an important and productive open-water region surrounded by sea ice). The authors demonstrate that phytoplankton carbon loss due to viral-mediated mortality was as important as grazing despite lower average specific viral lysis rates. The lysis of larger-sized key phytoplankton populations was largely responsible for this difference, which in turn has consequences for carbon export [33]. Marine ecosystems are under increasing pressure from warming, as well as climate-driven extreme weather and climate events [34]; thus, studies like those by Eich [32] and Woods [21] are key to understanding how ecosystems will respond to these alterations.

This SI highlights some of the multifaceted ways that viruses interact with plankton, from manipulating host metabolic pathways to causing significant fatalities, viruses shape plankton communities and drive biogeochemical cycling. In the face of global climate change, it is increasingly crucial to understand how virus–plankton interactions affect the flow of carbon and the role that viruses play in ecosystem stability and recovery.

## Figures and Tables

**Figure 1:**
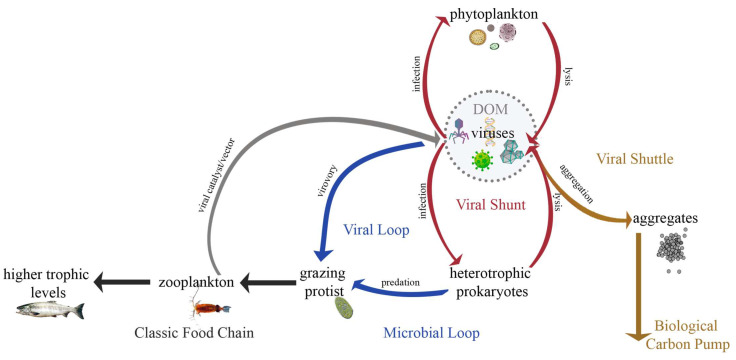
Schematic overview of plankton-virus interactions and their influence on the flow of carbon in aquatic environments.
